# Knockdown of TIPE2 increases the proliferation in lipopolysaccharide-stimulated gastric cancer cells

**DOI:** 10.1186/s12885-018-4761-3

**Published:** 2018-08-29

**Authors:** Wenming Liu, Yanyun Fan, Ying Shi, Zhenhe Lin, Xiaoxiao Huang, Wei Huang, Dongyan Shen, Zhongquan Qi

**Affiliations:** 1 Department of Gastroenterology, Zhongshan Hospital, Xiamen University, Xiamen, 361004 Fujian Province China; 2 Department of Gastroenterology, The First Affiliated Hospital, Jinan University, Guangzhou, 510632 Guangdong Province China; 3 The First Clinical Medical College, Jinan University, Guangzhou, 510632 Guanegdong Province China; 4 Organ Transplantation Institute, Medical College of Xiamen University, Xiamen, 361005 Fujian Province China; 5 Biobank, The First Affiliated Hospital of Xiamen University, Xiamen, 361003 Fujian Province China

**Keywords:** TIPE2, Gastric cancer, AKT, IκBα, Cell cycle, LPS

## Abstract

**Background:**

Gastric cancer (GC) is one of the most common malignant diseases with high morbidity and mortality, especially in Asian countries. During the GC developing progress, TIPE2, a member of TNF-alpha induced protein 8-like (TNFAIP8L) family, may play important roles. However, the molecular mechanisms of TIPE2 contributing to cell proliferation and tumor growth are poorly understood in GC. We performed flow cytometry to detect the cell cycle of TIPE2-knockdown GC cells under lipopolysaccharide (LPS) stimulation.

**Methods:**

We measured TIPE2 expression in tumor samples from 46 human GC patients at mRNA level by Realtime PCR and in 68 pairs of GC tissues at protein level by immunohistochemistry. We established stable TIPE2 knockdown SGC7901 and BGC823 cell lines and performed CCK-8 and EdU proliferation assays under the stimulation of LPS. And then we analyzed AKT, IκBα and ERK phosphorylation levels, as well as cycle related proteins CDK4 and CyclinD3 in the stable TIPE2 knockdown SGC7901 and BGC823 cells.

**Results:**

Our present studies indicated that the expression of TIPE2 was significantly decreased in tumor tissues compared to distant mucosa tissues in human GC patients. TIPE2 inhibited proliferation stimulated by LPS in SGC7901 and BGC823 cells. Silencing of TIPE2 significantly decreased cell G_0_/G_1_ phase ratio and increased G_2_/M phase. TIPE2 knockdown SGC7901 and BGC823 cells declined AKT and IκBα phosphorylation. TIPE2’s action on GC cell cycle was.

**Conclusions:**

Our results demonstrated that TIPE2 is a novel tumor suppressor gene that inhibits GC growth may mediated via AKT and IκBα phosphorylated activation. We revealed that TIPE2 may effectively interdict neoplasm development, which has potential clinical application values for GC targeted therapies.

## Background

Gastric cancer (GC) is one of the most common malignant diseases with high morbidity and mortality in digestive system [1, 2]. In China, new cases of GC accounted for 40% of the world incidence each year, which were ranked the second morbidity and the third mortality of the malignant tumors [3]. Currently, due to lack of specific and effective screening indicators, most patients have entered the advanced stage with poor treatment and poor prognosis [4].

TNFAIP8 expression in GC is correlated with tumor development and immune homeostasis [5]. TNFAIP8L1 enhances the apoptosis and plays an essential role in inflammatory associated tumorigenesis [6]. TNFAIP8L3 is an oncogene to promote carcinogenesis, highly expressing in human digestive tumor, such as colon and esophageal cancer [7, 8]. TIPE2, also known as TNFAIP8L2, negatively regulates both innate and adaptive immunity and decreases human hepatic cancer [6]. Deficiency of TIPE2 expression has also been found in non-small cell lung cancer (NSCLC) and renal cell carcinoma, which is associated with tumor metastasis and TNM staging [9, 10]. However, the molecular mechanisms of TIPE2 underlying GC initiation and development are still not explicit.

In our current study, we demonstrated that TIPE2 expression was down-regulated in GC progression. The effects and mechanism of TIPE2 in LPS-mediated proliferation and GC growth would be further discussed.

## Methods

### Ethics statement

This study was approved by the Ethics Committee of Zhongshan Hospital, Xiamen University (Xiamen, Fujian Province, China). Written consent was obtained from all the participants.

### Cell culture

The SGC7901 (TCHu 46) and BGC823 (TCHu 11) cells (purchased from Cell Bank, Shanghai Institutes for Biological Sciences, Shanghai, China) were cultured in RPMI 1640, with 10% fetal bovine serum (Life Technologies, Grand Island, NY, USA) and 1% penicillin G/streptomycin at 37 °C in an atmosphere of 95% air and 5% CO_2_.

### Establishment of stable TIPE2 knockdown cells

shRNA sequence 213 (5’-GATCCACCTGATCAAAGTGGCCATTTCAAGGAGAATGGCCACTTGATCAGGTTTTTTTACGCGTG-3′), sequence 431 (5′- GATCCGCAAGATCTGTGACGGACTTTCAAGAGAAGTCCGTCACAGATCTTGCTTTTTTTACGCGTG-3′), and sequence 523 (5′- GATCCGCATGACGGCACTTAGCTTTTCAAGAGAAAGCTAAGTGCCGTCATGGTTTTTTTACGCGTG -3′) for the *TIPE2* gene were selected using our own original algorithm.

### Western blot analysis

Cells with stable TIPE2 knockdown and the controls were incubated without or with LPS for 2 h, and then were analyzed by western blot. Primary antibodies against AKT (#4691), phospho-AKT (#4060), IκBα (#9242), phospho-IκBα (#2859), ERK (9102), phospho-ERK (9101) were purchased from Cell Signaling Technology (Boston, MA, USA). Primary antibody against TIPE2 (15940–1-AP), CDK4 (11026–1-AP), Cyclin D3 (26755–1-AP) and beta-actin (20536–1-AP) were purchased from Proteintech (Wuhan, Hubei, P.R.C).

### Immunohistochemical staining

GC tissue section was deparaffinized, rehydrated and then rinsed with PBS. High-pressure antigen retrieval was carried out in citrate antigen retrieval solution (MVS-0101; Maixin Biotech, Fuzhou, China). Endogenous peroxidase was blocked using endogenous peroxidase blocking solution (SP KIT-A1; Maixin Biotech, Fuzhou, China). The sections were incubated with 1% Triton X-100 for 10 min, followed by 10% normal donkey serum for 15 min at room temperature. Next, the sections were incubated overnight with rabbit anti-human TIPE2 antibody (15940–1-AP, Proteintech, Wuhan, Hubei, China) overnight at 4 °C. Next day, the sections were rinsed with PBS, incubated with biotin-labeled secondary antibody for 20 min at room temperature, rinsed againwith PBS, and incubatedwith horseradish peroxidase polymer conjugate (Elivision™ Super HRP (Mouse/Rabbit) IHC Kit-9922; Maixin Biotech, Fuzhou, China). The sections were stained with the chromogen 3,3-diaminobenzidine from the DAB Detection Kit (DAB-0031; Maixin Biotech, Fuzhou, China) for approximately 3 min and counterstained with hematoxylin.

### CCK-8 cell proliferation assay

Cells with stable TIPE2 knockdown and the controls were seeded into 96-well plates at a density of 2 × 10^3^ cells/well without or with 1 μg/ml LPS for 2 h. After 24, 48, 72, 96 h or 5 d, cells were incubated with Cell Counting Kit-8 solution (DoJinDo, Tokyo, Japan) for additional 1 h. The absorbance was measured using a microplate reader at a wavelength of 450 nm.

### EdU cell proliferation assay

The EdU assay was performed according to the manufacturers’ instructions (RiboBio). Cells were seeded at 2 × 10^4^ cells/well in a 6-well plate and then incubated with 1 μg/ml LPS for 2 h. Finally, cells were incubated with 50 μM EdU for 2 h, and then the nuclei were stained with DAPI (Invitrogen). The images were acquired using a Leica SP laser scanning microscope system.

### Flow cytometry (FCM) analysis

Cells were seeded at 1 × 10^4^ cells/well in a 12-well plate and then incubated with 1 μg/ml LPS for 2 h. Cell monolayers were collected and fixed by the dropwise addition of 70% ethanol at − 20 °C. Then, the fixed cells were washed with PBS and incubated in the dark for 30 min with 50 μg/ml propidium iodide (PI) and 100 μg/ml RNase A and measured with a flow cytometer (BD, Franklin Lakes, NJ) equipped with a 488-nm argon laser. Histograms of the PI intensities were plotted. The percentage of cells in each phase of the cell cycle was analyzed using ModFit software.

### Statistical analysis

Statistical analysis was performed using SPSS 21.0 software (SPSS Inc., Chicago, IL, USA). Student’s *t*-test (means ± standard deviation) and Chi-Square test were used for data analyze according to different data types. *p* <  0.05 or *p* <  0.01 was considered to be statistically significant. Graphs were illustrated by GraphPad Prism 5.0 (GraphPad Software Inc., La Jolla, CA, USA).

## Results

### TIPE2 expression is attenuated in GC tissues

**Fig. 1:**
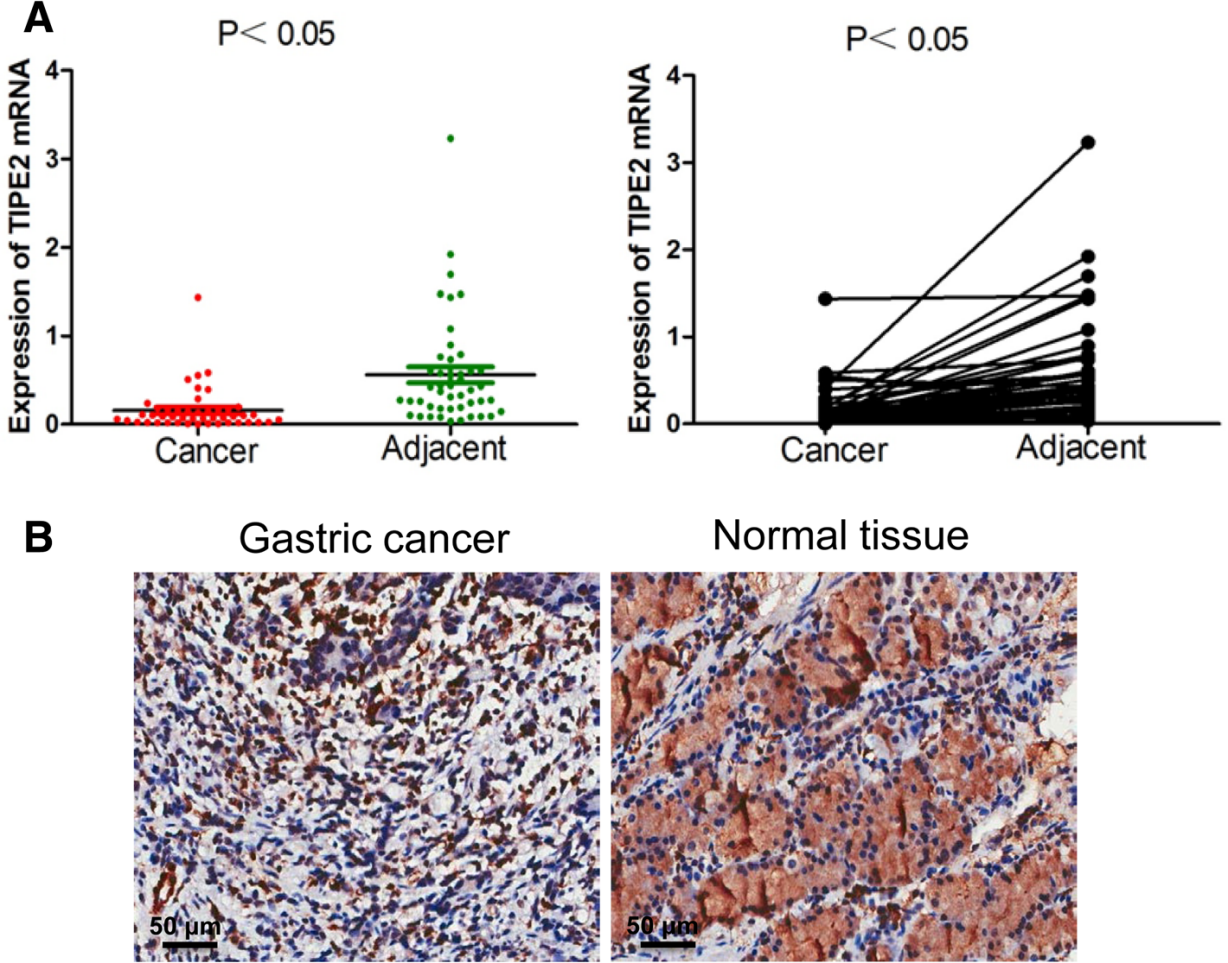
TIPE2 expression in human gastric cancer. **a** Tumor samples from 46 human GC patients were collected and detected. TIPE2 mRNA levels were significantly higher in human gastric cancer lesions than in adjacent cancerous tissues (*P* < 0.05). **b** TIPE2 protein levels were significantly lower in gastric cancer lesions than in adjacent tissues by immunohistochemistry

We collected tumor samples from 46 human GC patients and detected the expression in human GC tissues. TIPE2 expression at mRNA level was significantly lower in gastric tumor lesions than in adjacent non-cancerous tissues (Fig. 1a, *P* < 0.05). Consistent with mRNA levels, TIPE2 expression at protein levels were significantly lower in GC lesions than in adjacent tissues by immunohistochemistry (Fig. 1b). Additional samples were collected and TIPE2 expression was measured in 68 pairs of GC and normal tissue, the positive expression rate of TIPE2 in GC was 17.65%, significantly lower than that in normal gastric mucosa (72.06%) (Table 1, *P* < 0.00). Further analysis of clinical characteristics revealed that TIPE2 was closely associated with tumor differentiation, stages and lymph node metastasis. Low expression of TIPE2 indicated the worse differentiation and the poor prognosis of GC patients (Table 2). Thus, these results indicate that TIPE2 expression decreases as GC progresses.

**Table 1:** TIPE2 expression in 68 pairs of GC tissues

Group	TIPE2		Positive rate (%)	*p* value
	Positive	Negative		
Normal gastric mucosa	49	19	72.06	< 0.00
Gastric cancer	12	56	17.65	

In the 68 pairs of GC tissues, the positive expression rate of TIPE2 in GC and normal gastric mucosa. (*P* < 0.00)

**Table 2:** The clinical characteristics were analyzed

Characteristics	TIPE2		Positive rate (%)	*P* value
	Positive	Negative		
Gender				
Male	7	40	14.89	0.493
Female	5	16	23.81	
Age (yr)				
60 yr. or younger	3	16	15.79	1.000
Over 60 yr	9	40	18.37	
Differentiation				
Moderate	9	22	29.03	*0.024*
Poor	3	34	8.11	
Stage				
I + II	10	23	30.30	*0.008*
III + IV	2	33	5.71	
Lymph nodes involvement				
Yes	2	42	4.55	*0.000*
No	10	14	41.67	

TIPE2 was closely associated with tumor differentiation, stages and lymph node metastasis. *P* values with italics (*P* < 0.05) represented significant differences

### Knockdown of TIPE2 increases GC cell proliferation under the LPS stimulation in vitro

**Fig. 2:**
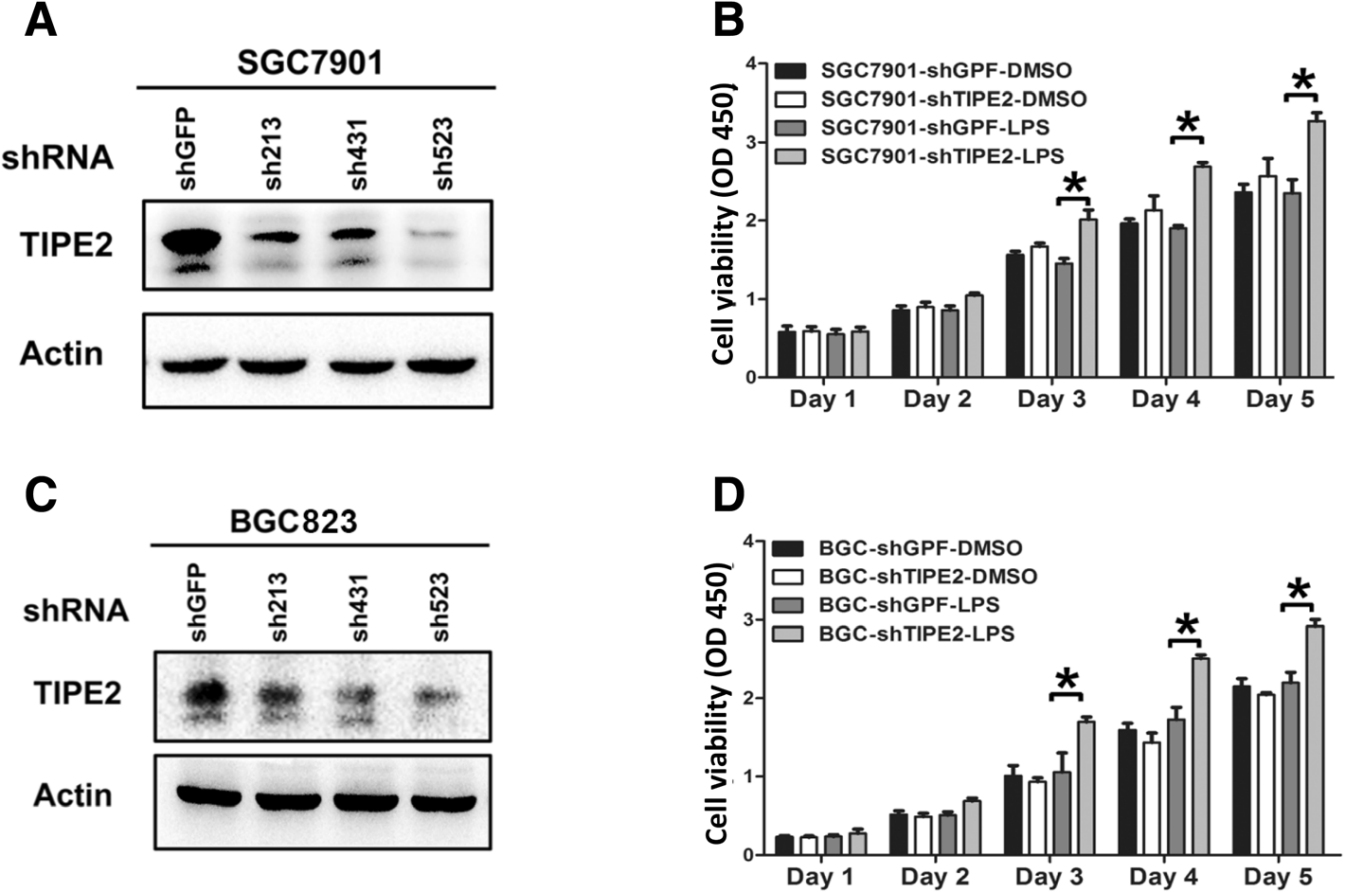
TIPE2 knockdown significantly increased the cell viability induced by LPS in GC cells. **a** and **c** The stable TIPE2 knockdown SGC7901 and BGC823 cell lines from three TIPE2 shRNA sequences were established (sh213, sh431 and sh523). **b** and **d** CCK-8 proliferation assays in SGC7901 (**b**) and BGC823 (**d**) cells showed significantly increased viability in TIPE2-knockdown cells after LPS treatment. All of the experiments were performed in triplicate. *P < 0.05

To determine the TIPE2 effects in GC development, we established stable TIPE2 knockdown SGC7901 and BGC823 cell lines from three TIPE2 shRNA sequences (sh213, sh431 and sh523), as well as controls. TIPE2 expression was drastically inhibited in the TIPE2 knockdown SGC7901 (Fig. 2a) and BGC823 (Fig. 2c) cell lines at protein level. Since sh523 showed higher efficacy than sh213 and sh431 in inhibiting TIPE2 expression, we chose sh523 for the subsequent experiments. Then we performed CCK-8 proliferation assays and demonstrated that there are no significant differences of cell proliferation ability between shGFP-control SGC7901 and BGC823 cells with or without 1 μg/ml lipopolysaccharide (LPS) treatment (Fig. 2b and d, shGFP-DMSO group versus shGFP-LPS group, DMSO as control). However, the cell proliferation was significantly increased in stable TIPE2 knockdown SGC7901 (Fig. 2b, *P* < 0.05) and BGC823 (Fig. 2d, *P* < 0.05) cells under the stimulation of LPS compared to the DMSO controls treatment.

**Fig. 3:**
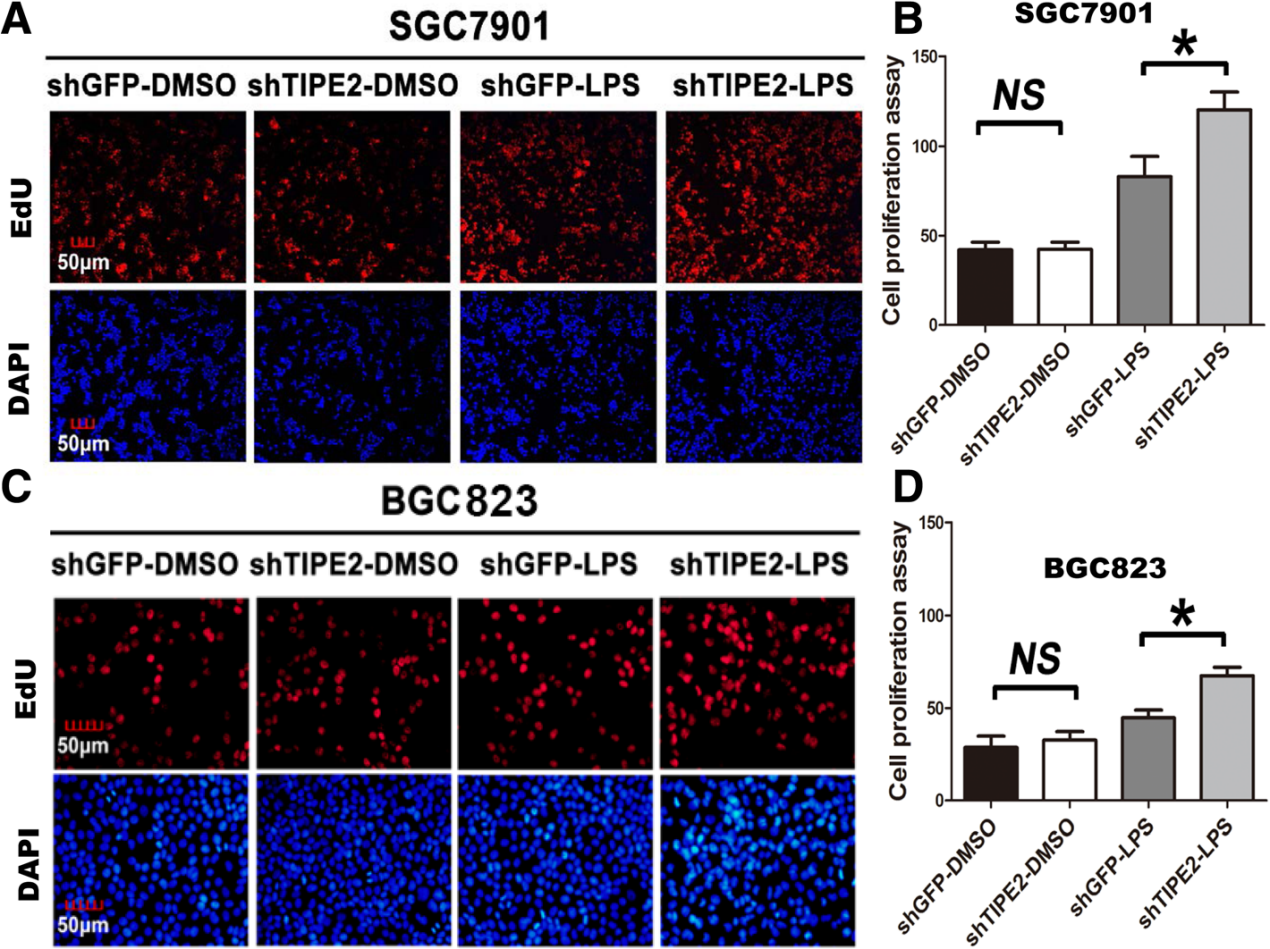
TIPE2 knockdown promotes proliferation of GC cells with LPS stimulation by EdU proliferation assays. **a** and **b** Treated with LPS, stable TIPE2-knockdown SGC7901 cells exhibited an increased cell proliferation than control cells. **c** and **d** Treated with LPS for 2 h, stable TIPE2-knockdown BGC823 cells exhibited an increased cell proliferation than their respective controls. Red represents EdU-positive cells and blue represents DAPI-stained DNA. All of the experiments were performed in triplicate. *P < 0.05. N.S. no significant differences

EdU proliferation assays were also performed in TIPE2-knockdown cell lines. Treated with LPS, stable TIPE2-knockdown SGC7901 and BGC823 cells showed increased cell proliferation compared with corresponding controls (Fig. 3a-d, *P* < 0.05). All these results collectively indicate that knockdown of TIPE2 promotes proliferation of GC cells under the LPS stimulation.

### Knockdown of TIPE2 promoting mitosis of GC cells stimulated by LPS

**Fig. 4:**
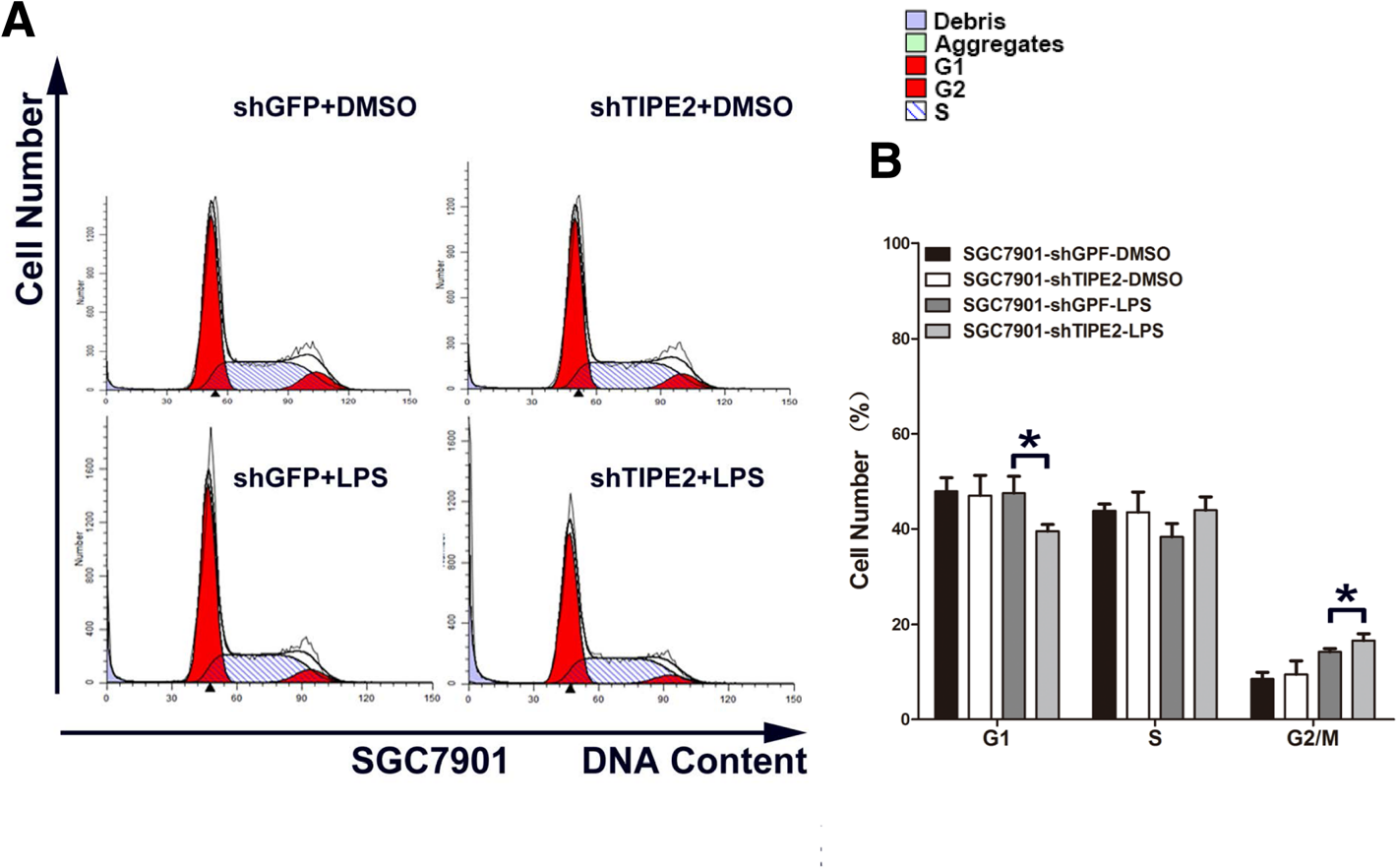
TIPE2 knockdown promoted mitosis of SGC7901 with LPS stimulation. **a** TIPE2 knockdown significantly decreased cell G_0_/G_1_ phase ratio and increased G_2_/M phase in SGC7901 cells by using flow cytometry. **b** The histograms of quantitative analysis. All of the experiments were performed in triplicate. *P < 0.05

**Fig. 5:**
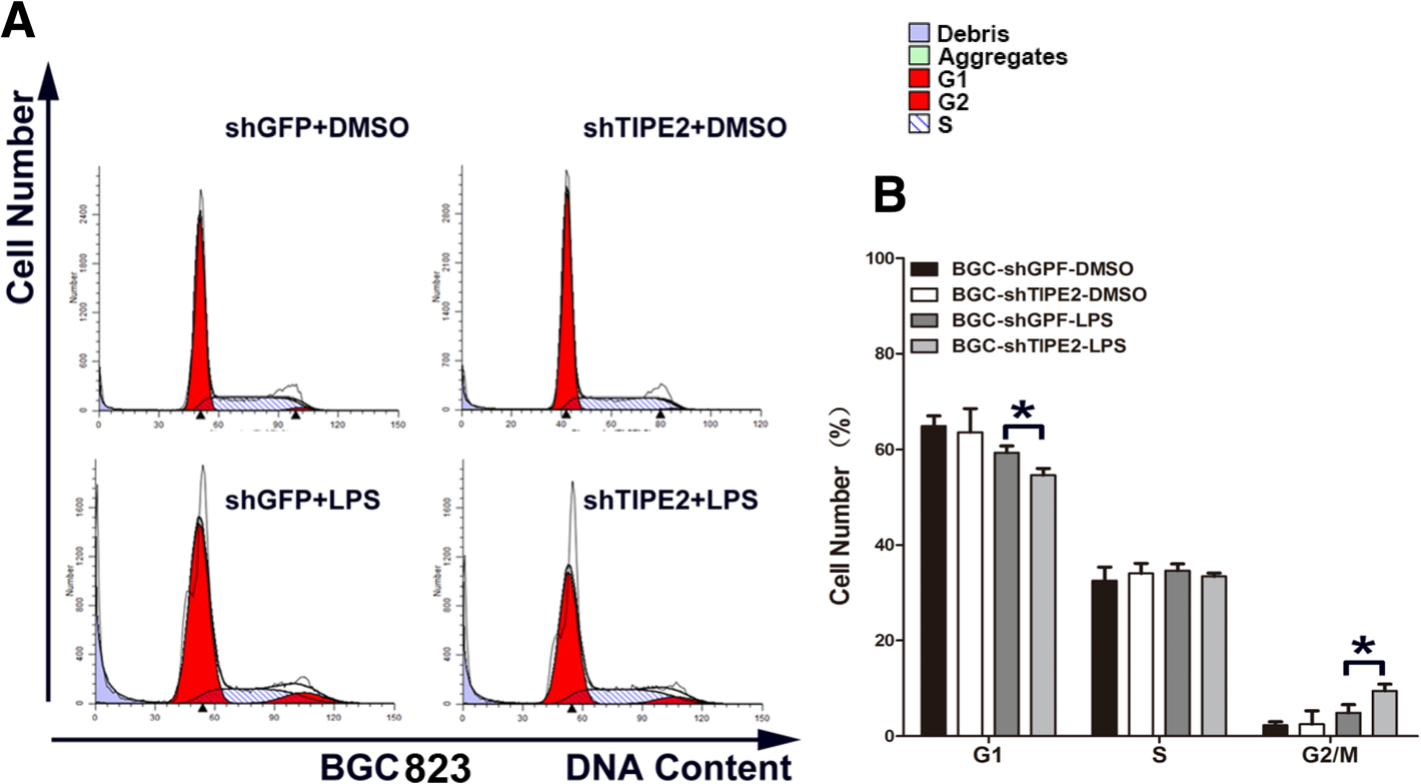
TIPE2 knockdown promoted mitosis of BGC823 with LPS stimulation. **a** TIPE2 knockdown significantly decreased cell G_0_/G_1_ phase ratio and increased G_2_/M phase in BGC823 cells by using flow cytometry. **b** The histograms of quantitative analysis. All of the experiments were performed in triplicate. *P < 0.05

Cell proliferation and cell cycle changes are closely related, thus, we performed flow cytometry to detect the cell cycle of TIPE2-knockdown GC cells under LPS stimulation. shTIPE2 significantly decreased cell G_0_/G_1_ phase ratio and increased G_2_/M phase in both SGC7901 (Fig. 4a and b) and BGC823 (Fig. 5a and b). Thus, shTIPE2 promoted mitosis of GC cells to increase cell proliferation.

### Knockdown TIPE2 activates AKT and IκBα phosphorylation in GC cells

**Fig. 6:**
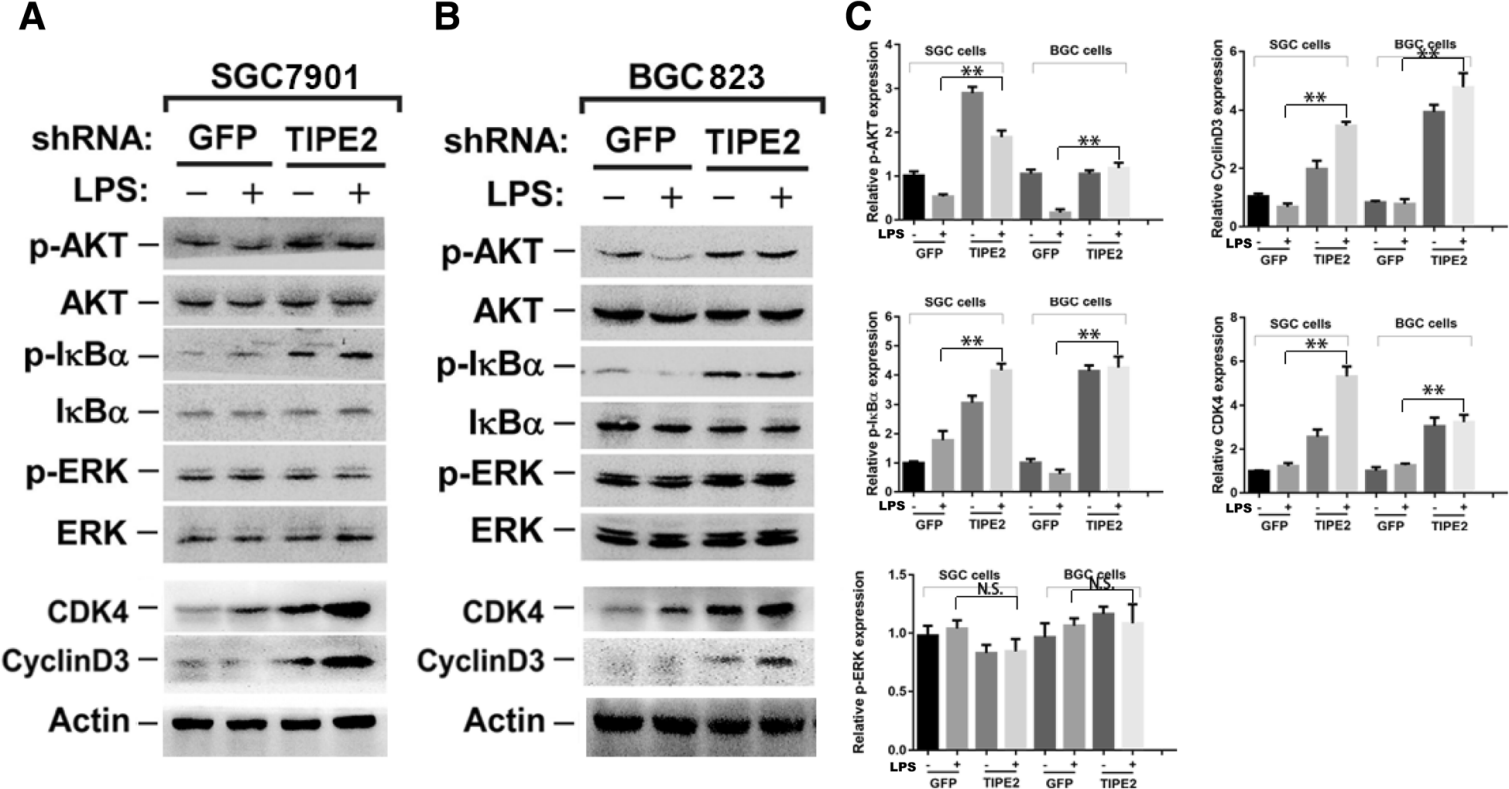
LPS stimulated TIPE2 knockdown cells show increased levels of pAKT and pIkBa as well as CyclinD3 and CDK4. **a** and **b** AKT, IκBα and ERK phosphorylation levels were analyzed in the stable TIPE2 knockdown SGC7901 and BGC823 cells. AKT and IκBα phosphorylation was up-regulated in the TIPE2 knockdown SGC7901 and BGC823 cells, while no significant differences in ERK phosphorylation were observed. The cell cycle related proteins were examined. CDK4 and CyclinD3 levels were significantly upregulated in TIPE2 knockdown SGC7901 and BGC823 cells compared with control cells. **c** The histograms of quantitative analysis. All of the experiments were performed in triplicate. *P < 0.05

AKT, IκBα and ERK phosphorylation levels were analyzed to identify the molecular signaling pathways of TIPE2-mediated GC cell proliferation. AKT and IκBα phosphorylation was declined in TIPE2 knockdown SGC7901 and BGC823 cells, while no significant differences in ERK phosphorylation were observed (Fig. 6a-d).

Next, we examined cell cycle related proteins and explored that CDK4 and CyclinD3 levels were significantly upregulated in TIPE2 knockdown SGC7901 and BGC823 cells compared with control cells (Fig. 6a-d). Therefore, TIPE2 regulates the proliferation may via AKT and IκBα phosphorylated activation in GC cells.

## Discussion

TIPE family contains three members, TIPE1, TIPE2 and TIPE3. The TIPE1 protein is located in the cytoplasm, and the gene expressed in hepatocytes, neurons, muscular tissues, reproductive organs and epithelial cells [5]. TIPE3 expressed in human gastrointestinal and endocrine systems, as well as in hemicerebrum [8]. TIPE1 and TIPE3 plays crucial effect on carcinogenesis and cell secretion [6, 7]. TIPE2 negatively regulates the inflammation and immune homeostasis by restraining T cell receptor (TCR) and Toll-like receptor (TLR) signaling [5]. TIPE2 also expresses in autoimmune hepatitis and chronic hepatitis B [11, 12]. Hepatitis C virus augments hepatocellular tumorigenesis by targeting TIPE2 [13]. TIPE2 expression increases in peripheral blood mononuclear cells (PBMCs), but decreased in hyperstretched bronchial epithelial cells [14, 15].

TIPE2 also closely related with malignant diseases. TIPE2 suppresses the proliferation and tumorigenesis by inhibiting β-catenin, cyclin D1 and c-Myc, and metastasis via AKT and p38 signaling pathways in breast cancer [16–18]. Besides, TIPE2 impedes the prostate cancer progression, [19], inhibits hypoxia-induced epithelial mesenchymal transition (EMT) in glioma cells [20], and upregulated in non-hodgkin’s lymphoma [21].

However, the functions of TIPE2 in gastrointestinal malignant cancer are still unclear. It has been reported that TIPE2 inhibits GC metastasis by EMT reversal [22]. In our current studies, we display that TIPE2 expression was decreased in GC tissues compared to control tissues in human GC patients. TIPE2 inhibited proliferation stimulated by LPS in SGC7901 and BGC823 GC cells. Knockdown of TIPE2 up-regulated AKT and IκBα phosphorylation in SGC7901 and BGC823 cells, while no significant differences in ERK phosphorylation were observed. The cell cycle related proteins CDK4 and CyclinD3 levels were examined to significantly up-regulate in TIPE2 knockdown SGC7901 and BGC823 cells compared with control cells. Knockdown of TIPE2’s action on GC cell cycle was mediated via AKT and IκBα phosphorylated activation. Therefore, we indicated that TIPE2 was a tumor suppressor gene that inhibited GC growth in an AKT and IκBα dependent manner.

In conclusion, our present study reveals that knockdown of TIPE2 mediated GC growth may via AKT and IκBα phosphorylated activation stimulates by LPS. TIPE2 knockdown upregulates cell cycle related proteins CDK4 and CyclinD3. TIPE2 may be used as a potential therapeutic strategy for GC therapy.

## Conclusions

In summary, our present studies demonstrated that TIPE2 is a novel tumor suppressor gene that inhibits GC growth. We revealed that TIPE2 may effectively interdict neoplasm development, which has potential clinical application values for GC targeted therapies.

## Abbreviations

GC: Gastric cancer
LPS: Lipopolysaccharide
TNFAIP8L: TNF-alpha induced protein 8-like

## References

[1] Rugge M, Genta RM, Graham DY, Di Mario F, Vaz Coelho LG, Kim N, Malfertheiner P, Sugano K, Tsukanov V, Correa P (2016). Chronicles of a cancer foretold: 35 years of gastric cancer risk assessment. Gut.

[2] Siegel RL, Miller KD, Jemal A (2018). Cancer statistics, 2018. CA Cancer J Clin.

[3] Chen W, Zheng R, Zhang S, Zhao P, Li G, Wu L, He J (2013). The incidences and mortalities of major cancers in China, 2009. Chin J Cancer.

[4] Cancer Genome Atlas Research N (2014). Comprehensive molecular characterization of gastric adenocarcinoma. Nature.

[5] Lou Y, Liu S (2011). The TIPE (TNFAIP8) family in inflammation, immunity, and cancer. Mol Immunol.

[6] Goldsmith JR, Chen YH. Regulation of inflammation and tumorigenesis by the TIPE family of phospholipid transfer proteins. Cell Mol Immunol. 2017;14(6):482–7. 10.1038/cmi.2017.4 PMC5518821 28287114

[7] Fayngerts Svetlana A, Wu J, Oxley Camilla L, Liu X, Vourekas A, Cathopoulis T, Wang Z, Cui J, Liu S, Sun H (2014). TIPE3 is the transfer protein of lipid second messengers that promote Cancer. Cancer Cell.

[8] Cui J, Hao C, Zhang W, Shao J, Zhang N, Zhang G, Liu S (2015). Identical expression profiling of human and murine TIPE3 protein reveals links to its functions. J Histochem Cytochem.

[9] Li Z, Guo C, Liu X, Zhou C, Zhu F, Wang X, Wang Q, Shi Y, Wang J, Zhao W (2016). TIPE2 suppresses angiogenesis and non-small cell lung cancer (NSCLC) invasiveness via inhibiting Rac1 activation and VEGF expression. Oncotarget.

[10] Zhang Z, Qi H, Hou S, Jin X (2013). TIPE2 mRNA overexpression correlates with TNM staging in renal cell carcinoma tissues. Oncol Lett.

[11] Qian J, Meng Z, Guan J, Zhang Z, Wang Y (2017). Expression and roles of TIPE2 in autoimmune hepatitis. Exp Ther Med.

[12] Fan YC, Zhang YY, Wang N, Sun YY, Wang K. Tumor necrosis factor-alpha-induced protein 8-like 2 (TIPE2) is associated with immune phases of patients with chronic hepatitis B. Oncotarget. 2017;8(19):30781–92. 10.18632/oncotarget.15683 PMC5458167 28390195

[13] Wang Y, Jiang Y, Zhou J, Song W, Li J, Wang M, Chen J, Xu R, Zhang J, Ma F (2016). Hepatitis C virus promotes hepatocellular carcinogenesis by targeting TIPE2, a new regulator of DNA damage response. Tumour Biol.

[14] Zhao P, Wang L, Xiang X, Zhang X, Zhai Q, Wu X, Li T (2017). Increased expression of TIPE2 mRNA in PBMCs of patients with ankylosing spondylitis is negatively associated with the disease severity. Hum Immunol.

[15] Sun X, Chen L, Yan W. TIPE2 inhibits the expression of asthma-related inflammatory factors in Hyperstretched bronchial epithelial cells through the Wnt/beta-catenin pathway. Inflammation. 2017;40(3):770–77. 10.1007/s10753-017-0521-9 28188409

[16] Wang K, Ren Y, Liu Y, Zhang J, He JJ (2017). Tumor necrosis factor (TNF)-alpha-induced protein 8-like-2 (TIPE2) inhibits proliferation and tumorigenesis in breast Cancer cells. Oncol Res.

[17] Zhang Z, Liu L, Liu C, Cao S, Zhu Y, Mei Q (2016). TIPE2 suppresses the tumorigenesis, growth and metastasis of breast cancer via inhibition of the AKT and p38 signaling pathways. Oncol Rep.

[18] Zhang Z, Liu L, Cao S, Zhu Y, Mei Q (2017). Gene delivery of TIPE2 inhibits breast cancer development and metastasis via CD8+ T and NK cell-mediated antitumor responses. Mol Immunol.

[19] Lu Q, Liu Z, Li Z, Chen J, Liao Z, Wu WR, Li YW (2016). TIPE2 overexpression suppresses the proliferation, migration, and invasion in prostate Cancer cells by inhibiting PI3K/Akt signaling pathway. Oncol Res.

[20] Liu ZJ, Liu HL, Zhou HC, Wang GC (2016). TIPE2 inhibits hypoxia-induced Wnt/beta-catenin pathway activation and EMT in glioma cells. Oncol Res.

[21] Hao C, Zhang N, Geng M, Ren Q, Li Y, Wang Y, Chen YH, Liu S (2016). Clinical significance of TIPE2 protein upregulation in non-Hodgkin's lymphoma. J Histochem Cytochem.

[22] Yin H, Huang X, Tao M, Hu Q, Qiu J, Chen W, Wu J, Xie Y (2017). Adenovirus-mediated TIPE2 overexpression inhibits gastric cancer metastasis via reversal of epithelial-mesenchymal transition. Cancer Gene Ther.

